# The effect of sleep restriction, with or without high‐intensity interval exercise, on myofibrillar protein synthesis in healthy young men

**DOI:** 10.1113/JP278828

**Published:** 2020-03-11

**Authors:** Nicholas J. Saner, Matthew J.‐C. Lee, Nathan W. Pitchford, Jujiao Kuang, Gregory D. Roach, Andrew Garnham, Tanner Stokes, Stuart M. Phillips, David J. Bishop, Jonathan D. Bartlett

**Affiliations:** ^1^ Institute for Health and Sport Victoria University Melbourne Australia; ^2^ School of Medical & Health Sciences Edith Cowan University Joondalup Australia; ^3^ Department of Kinesiology McMaster University Hamilton Canada; ^4^ Sport Performance Optimisation Research Team School of Human Life Sciences University of Tasmania Launceston Australia; ^5^ Appleton Institute for Behavioural Science Central Queensland University Adelaide Australia

**Keywords:** atrophy, high‐intensity interval exercise, protein synthesis, sleep loss

## Abstract

**Key points:**

- Sleep restriction has previously been associated with the loss of muscle mass in both human and animal models.
- The rate of myofibrillar protein synthesis (MyoPS) is a key variable in regulating skeletal muscle mass and can be increased by performing high‐intensity interval exercise (HIIE), although the effect of sleep restriction on MyoPS is unknown.
- In the present study, we demonstrate that participants undergoing a sleep restriction protocol (five nights, with 4 h in bed each night) had lower rates of skeletal muscle MyoPS; however, rates of MyoPS were maintained at control levels by performing HIIE during this period.
- Our data suggest that the lower rates of MyoPS in the sleep restriction group may contribute to the detrimental effects of sleep loss on muscle mass and that HIIE may be used as an intervention to counteract these effects.

**Abstract:**

The present study aimed to investigate the effect of sleep restriction, with or without high‐intensity interval exercise (HIIE), on the potential mechanisms underpinning previously‐reported sleep‐loss‐induced reductions to muscle mass. Twenty‐four healthy, young men underwent a protocol consisting of two nights of controlled baseline sleep and a five‐night intervention period. Participants were allocated into one of three parallel groups, matched for age, ${\dot{V}}_{O_{2\;\mathrm{peak}}}$, body mass index and habitual sleep duration; a normal sleep (NS) group [8 h time in bed (TIB) each night], a sleep restriction (SR) group (4 h TIB each night), and a sleep restriction and exercise group (SR+EX, 4 h TIB each night, with three sessions of HIIE). Deuterium oxide was ingested prior to commencing the study and muscle biopsies obtained pre‐ and post‐intervention were used to assess myofibrillar protein synthesis (MyoPS) and molecular markers of protein synthesis and degradation signalling pathways. MyoPS was lower in the SR group [fractional synthetic rate (% day^–1^), mean ± SD, 1.24 ± 0.21] compared to both the NS (1.53 ± 0.09) and SR+EX groups (1.61 ± 0.14) (*P *< 0.05). However, there were no changes in the purported regulators of protein synthesis (i.e. p‐AKT^ser473^ and p‐mTOR^ser2448^) and degradation (i.e. *Foxo1/3* mRNA and LC3 protein) in any group. These data suggest that MyoPS is acutely reduced by sleep restriction, although MyoPS can be maintained by performing HIIE. These findings may explain the sleep‐loss‐induced reductions in muscle mass previously reported and also highlight the potential therapeutic benefit of HIIE to maintain myofibrillar remodelling in this context.

## Key points

- Sleep restriction has previously been associated with the loss of muscle mass in both human and animal models.
- The rate of myofibrillar protein synthesis (MyoPS) is a key variable in regulating skeletal muscle mass and can be increased by performing high‐intensity interval exercise (HIIE), although the effect of sleep restriction on MyoPS is unknown.
- In the present study, we demonstrate that participants undergoing a sleep restriction protocol (five nights, with 4 h in bed each night) had lower rates of skeletal muscle MyoPS; however, rates of MyoPS were maintained at control levels by performing HIIE during this period.
- Our data suggest that the lower rates of MyoPS in the sleep restriction group may contribute to the detrimental effects of sleep loss on muscle mass and that HIIE may be used as an intervention to counteract these effects.

## Introduction

Sleep plays a key role in many physiological and cognitive functions, and it is recommended that adults obtain between 7 and 9 h of sleep each night (Spiegel *et al*. 1999; Van Dongen *et al*. 2003; National Sleep Foundation, 2015). Sleep has also been causatively implicated in the maintenance of muscle mass (Nedeltcheva *et al*. 2010; Dattilo *et al*. 2012), with an increased likelihood of both sarcopenia and lower total skeletal muscle mass in those reporting insufficient (i.e. <7 h per night) or poor sleep quality (Chien *et al*. 2015; Buchmann *et al*. 2016; Hu *et al*. 2017). During periods of energy deficit, a disproportionately greater loss of muscle mass has been reported in humans following sleep restriction [5.5 h time in bed (TIB) each night for 14 days] compared to normal sleep (8.5 h TIB each night for 14 days) (Nedeltcheva *et al*. 2010). In animal models, muscle atrophy has also been observed following sleep deprivation (i.e. >24 h of extended wakefulness) (Dattilo *et al*. 2012; Monico‐Neto *et al*. 2015*a*; Monico‐Neto *et al*. 2015*b*; de Sa Souza *et al*. 2016); however, the potential mechanisms by which sleep restriction may lead to muscle atrophy have not been comprehensively studied in humans.

Changes in muscle mass are, in the short‐term (i.e. days to weeks), largely determined by the balance between the rates of muscle protein synthesis (MPS) and muscle protein breakdown (MPB) (Rennie, 1985; Gibson *et al*. 1987). Previous reports suggest that sleep deprivation may influence these processes. Indeed, protein catabolism in humans (determined by urea excretion) was increased following 72 h of sleep deprivation (Kant *et al*. 1984), although whether this was accompanied by changes in muscle protein turnover is unknown. Interventions known to induce loss of muscle mass (such as muscle disuse, limb immobilization and step reduction) suggest that reductions in MPS, and specifically myofibrillar protein synthesis (MyoPS), rather than increases in MPB, underlie these changes (Symons *et al*. 2009; Breen *et al*. 2013; Rudrappa *et al*. 2016). Accordingly, a reduction in MPS, and potentially MyoPS, may underpin reductions to muscle mass induced by sleep restriction. However, a potential catabolic influence of sleep restriction cannot be excluded given previous findings of increased autophagy and ubiquitination markers in rodent sleep deprivation studies (Monico‐Neto *et al*. 2015*a*; de Sa Souza *et al*. 2016). Nonetheless, no study to date has investigated the effects of sleep restriction on MPS, in whole or fractionated human skeletal muscle, or the potential catabolic influence of sleep restriction on markers of MPB.

At the molecular level, protein synthesis is regulated via the activation of the AKT‐mTOR‐p70S6K signalling pathway, which subsequently leads to increases in strength and muscle mass over time (Bodine *et al*. 2001). However, little is known about how sleep restriction may influence these pathways. Following 96 h of sleep deprivation, either no change in p‐mTOR^ser2448^ and p‐p70S6K^thr389^ (Monico‐Neto *et al*. 2015*a*) or an increase in p‐mTOR^ser2448^ and p‐4EBP1^ser65^ (de Sa Souza *et al*. 2016) were observed in rodent skeletal muscle. Although MPS is considered the driving force underpinning changes in muscle mass, MPB may also contribute to these changes. Molecular markers of the ubiquitin proteasome system (UPS) (i.e. FOXO1/3, MuRF1 and MAFbx), as well as the autophagy signalling pathways (i.e. LC3 and p62/SQSTM1) that lead to protein degradation, are often measured as a proxy for protein degradation (Tipton *et al*. 2018). In rodents, 96 h of sleep deprivation elevated levels of ubiquitinated proteins, markers of protein degradation (e.g. p‐FOXO3^Ser253^) and autophagy pathways (e.g. LC3 and p62/SQSTM1) and also induced muscle atrophy (Monico‐Neto *et al*. 2015*a*; de Sa Souza *et al*. 2016). However, such mechanisms in human models reflecting levels of sleep loss commonly experienced in society are yet to be investigated.

Although resistance exercise is regarded as the most potent stimulus to induce skeletal muscle protein synthesis, high‐intensity interval exercise (HIIE) is also capable of inducing muscle protein synthesis, and its associated signalling pathways (Miller *et al*. 2005; Di Donato *et al*. 2014; Bell *et al*. 2015). Given the increasing prevalence of inadequate sleep in modern society (Ford *et al*. 2015) and the important metabolic and structural roles of skeletal muscle, there are clear health implications to mitigating reductions in muscle mass resulting from inadequate sleep. Given the potency of the stimulus provided by HIIE to stimulate MyoPS (Bell *et al*. 2015) and its time‐efficient nature (Hood *et al*. 2011), as well as its other proven clinical benefits (such as improvements in insulin sensitivity and oxidative capacity) (Little *et al*. 2011; Saner *et al*. 2018), HIIE may be a potential therapeutic intervention to counteract the detrimental effects of sleep restriction on muscle quality.

The effects of sleep restriction and HIIE on MyoPS and its associated signalling pathways have not previously been investigated in humans. Consequently, the present study aimed to determine the potential mechanisms by which sleep restriction may contribute to previously reported reductions in muscle mass. Specifically, the study used a previously published model of sleep restriction (i.e. five nights, with 4 h TIB each night) (Reynolds *et al*. 2012) to investigate the effect of sleep restriction, with or without three sessions of HIIE, on the rate of MyoPS and associated molecular signalling pathways. We hypothesized that the rate of MyoPS would be lower in the sleep restriction group and this would be reflected by changes in the molecular signalling pathways associated with MPS and MPB, whereas performing three sessions of HIIE would attenuate this effect.

## Methods

### Ethical approval

All procedures involved conform to the standards set by the latest revision of the *Declaration of Helsinki* (except for registration in a database) and were approved by the Victoria University Human Research Ethics Committee (HRE15‐294).

### Participants

Twenty‐four healthy, recreationally‐active men, aged between 18 and 40 years of age, volunteered to participate, having had all procedures and risks explained, and provided their written informed consent. Participants were screened to determine their eligibility, as well as to rule out any pre‐existing medical conditions that may have precluded their participation (e.g. cardiovascular, metabolic or musculoskeletal problems). Eligible participants included in the study were (i) not taking any medications before and during the study; (ii) not performing shift work (within the previous three months); (iii) had regular sleeping habits and no previously‐diagnosed sleep disorders; (iv) had not travelled overseas in the previous two months; and (v) had a body mass index between 19 and 30 kg m^–2^. Prior to commencing the study, 1 week of habitual sleep was monitored via wristwatch actigraphy and sleep diaries; participants who averaged <6 or >9 h sleep per night during the monitoring period were also excluded from the study.

### Study setting

The study was conducted in a temperature‐controlled sleep laboratory with the capacity for three participants to complete the study at any one time, each within their own bedroom. Participants were monitored at all times throughout the study by a member of the research team to ensure adherence to the protocol.

### Study overview

Following the initial screening procedures, eligible participants attended the exercise physiology laboratory for baseline assessments of anthropometric measurements (i.e. height and body mass), and aerobic fitness (${\dot{V}}_{O_{2\;\mathrm{peak}}}$) determined from a graded exercise test (GXT). In a counterbalanced order, participants were then assigned to one of three experimental groups, matched for age, body mass index, habitual sleep duration, and ${\dot{V}}_{O_{2\;\mathrm{peak}}}$: normal sleep (NS, *n *= 8), sleep restriction (SR, *n *= 8) or sleep restriction and exercise (SR+EX, *n *= 8). Baseline participant characteristics for the three groups are shown in Table 1. One week prior to beginning the study participants also provided a pre‐study, resting skeletal muscle biopsy, to determine baseline levels of deuterium oxide (D_2_O) enrichment.

**Table 1:** Baseline characteristics of participants

	NS (*n* = 8)	SR (*n* = 8)	SR+EX (*n* = 8)
Age (years)	24 ± 4	25 ± 5	24 ± 4
Height (cm)	177 ± 8	179 ± 6	179 ± 7
Mass (kg)	78.7 ± 13.3	74.5 ± 11.7	80.2 ± 9.5
BMI	25.2 ± 3.6	23.3 ± 3.0	24.6 ± 2.5
${\dot{V}}_{O_{2\;\mathrm{peak}}}$ (mL kg^−1^ min^−1^)	43.7 ± 9.7	47.2 ± 6.7	48.0 ± 5.0
${\dot{W}}_{\mathrm{Peak}}$ (W)	319 ± 59	330 ± 44	362 ± 48
Habitual sleep duration (min)	457 ± 45	428 ± 44	437 ± 39

Values are the mean ± SD. There were no statistically significant differences between the three groups for any of the baseline characteristics. BMI, body mass index.

**Figure 1:**
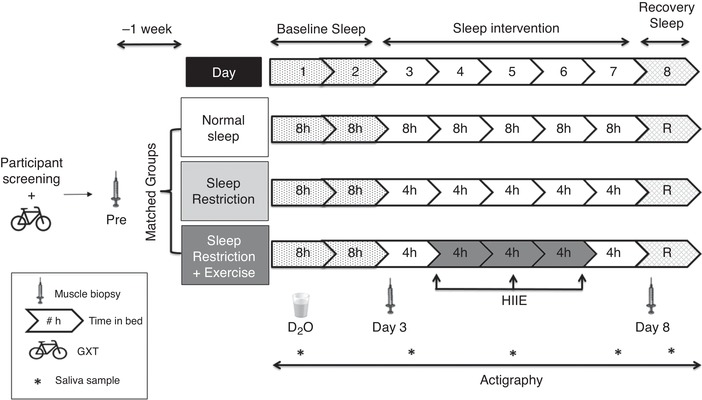
Schematic representation of the study protocol D_2_O – deuterium oxide ingestion; R, *ad libitum* recovery sleep; participant screening refers to medical questionnaires, exclusion criteria and habitual sleep, as well as physical activity monitoring.

The experimental component of the study consisted of an eight‐night stay within the sleep laboratory. All groups completed two initial nights of baseline sleep (8 h TIB, 23.00 h to 07.00 h), followed by a five‐night intervention period, during which the NS group spent 8 h TIB (23.00 h to 07.00 h), whereas both SR and SR+EX spent 4 h TIB per night (03.00 h to 07.00 h). Between 23.00 h and 03.00 h, lighting was dimmed to below 15 lux to reduce the effect of lighting on circadian rhythms (Duffy & Wright, 2005). The SR+EX group also performed three exercise sessions during the intervention period on days 4, 5 and 6 at 10.00 h. Following the intervention period, all groups completed a final night of *ad libitum* recovery sleep. An overview of the study protocol is shown in Fig. 1.

To assess MyoPS and molecular markers of protein synthesis and degradation pathways, two additional resting skeletal muscle biopsies were sampled during the experimental testing sessions, at 10.00 h on both day 3 (following two nights of baseline sleep) and day 8 (following the final night of the sleep intervention). Throughout the study, participants were provided with a standardized diet consisting of fixed proportions (relative to body mass) of carbohydrates (4.5 g kg^−1^ day^−1^), protein (1.5 g kg^−1^ day^−1^) and fat (1 g^ ^kg^−1.^d^−1^). All meal times (six throughout the day) were kept constant throughout the study and participants were requested to eat all food provided. Dietary intake was replicated on the days prior to both experimental sessions. Caffeine intake was prohibited throughout the study. Participants were asked to match their habitual step counts by walking outside of the facility at designated periods throughout the day, accompanied by a member of the research staff. Waking hours were spent watching television, reading, working on a computer or talking to the staff.

### Experimental procedures

#### Sleep monitoring

Sleep was assessed using wrist‐watch activity devices worn on the non‐dominant wrist (Actiwatch 2; Philips Respironics, Murrysville, PA, USA) with device activity sensitivity set to medium (40 activity counts per 30 s epoch) (Kosmadopoulos *et al*. 2014; Sargent *et al*. 2016), collected at 32 Hz and scored using a validated automated scoring algorithm (Oakley, 1997). These devices were used to objectively measure total sleep time (TST) obtained both habitually (prior to the study) and throughout the study. A member of the research team recorded the exact time that bedroom lights were switched on and off, allowing participants the designated sleep opportunity each night.

#### Physical activity monitoring

Throughout the study, and during 1 week prior (for habitual step‐count), daily step counts were monitored using commercially available and validated step‐counting applications on the participants’ personal mobile phone devices (i‐Health app; Apple Inc., Cupertino, CA, USA; and Samsung Health, Samsung Electronics Co., Ltd., Suwon, South Korea) (Hochsmann *et al*. 2018). Participants were encouraged to replicate their habitual daily step counts throughout the study protocol (Table 2). Participants were supervised at all times, and instructed to avoid any additional moderate or vigorous physical activity throughout the study.

**Table 2:** Habitual and sleep study step counts for participants in each group

Step count	NS	SR	SR+EX
Habitual	12260 ± 3964	10965 ± 2136	11831 ± 919
Study	10652 ± 2476	10033 ± 1839	10953 ± 2316

Values are the mean ± SD. There were no significant differences between daily step counts, habitually and within the study protocol, in any of the groups.

#### GXT

A baseline assessment of aerobic fitness (i.e. peak oxygen uptake (${\dot{V}}_{O_{2\;\mathrm{peak}}}$) and peak power (W) (${\dot{W}}_{\mathrm{Peak}}$) was performed on an electronically‐braked cycle ergometer (Excalibur, V2.0; Lode, Groningen, The Netherlands). Following a standardized warm‐up (5 min at 30 W), participants cycled against an incremental ramp protocol where the resistance continually increased by 1 W every 2 s (30 W min^−1^) until volitional exhaustion (i.e. cadence fell below 60 rpm). Expired air was continuously analysed for O_2_ and CO_2_ concentrations via a gas analyser (Moxus 2010; AEI Technologies, Pittsburgh, PA, USA), which was calibrated immediately before each test.

#### HIIE

The HIIE protocol was adapted from a previous study (Little *et al*. 2010) and consisted of 10 × 60 s intervals performed on a cycle ergometer (RacerMate; Velotron, Seattle, WA, USA) at 90% of each participant's ${\dot{W}}_{\mathrm{peak}}$. Each interval was interspersed with 75 s of active recovery (at 60 W). Each session started with a 3 min warm up at 60 W. Heart rate (HR) (FT1; Polar, Kempele, Finland) and ratings of perceived exertion (RPE) (Borg, 1970) were recorded at the end of each interval. The mean power per interval was 318 ± 53 W, mean HR throughout the protocol was 156 ± 13 bpm, peak HR was 182 ± 12 bpm, and the average RPE per interval was 15 ± 2 AU.

#### Muscle biopsies

One week prior to commencing the study, and during the experimental sessions (day 3 and day 8), muscle biopsies were sampled from the vastus lateralis muscle of the same leg, using a suction‐modified Bergström needle, under local anaesthesia of the skin and fascia (1% lidocaine). The pre‐study biopsy was sampled at 10.00 h, following an overnight fast, and was used to determine background enrichment of D_2_O (prior to the ingestion of the D_2_O bolus) for the subsequent MyoPS analysis. The samples collected on days 3 and 8 were also obtained at 10.00 h, both following an overnight fast. All samples were immediately frozen in liquid nitrogen and stored at –80°C for subsequent analyses.

#### Assessment of MyoPS

##### D_2_O ingestion and saliva sample collection

The fractional synthetic rate (FSR) of MyoPS was assessed via the D_2_O tracer technique as previously reported (Wilkinson *et al*. 2014). At 18.00 h on day 1, each participant ingested 150 mL of D_2_O (70 atom %; Cambridge Isotope Laboratories, Tewksbury, MA, USA). Saliva samples were collected in salivettes prior to D_2_O ingestion and then on days 3, 5, 7 and 8 to determine body water enrichment. ^2^H enrichment of saliva was determined by a cavity ring‐down spectroscopy with the use of a liquid isotope analyser (Picarro L2130‐I analyser; Picarro, Santa Clara, CA, USA) with an automated injection system. Total body water ^2^H enrichment was used as a surrogate for plasma alanine ^2^H labelling, as previously described (Wilkinson *et al*. 2014). The mean body water enrichment [atomic percentage excess (APE), mean ± SD) throughout the intervention was 0.18 ± 0.03 APE on day 3, 0.15 ± 0.04 APE on day 5, 0.12 ± 0.03 APE on day 7 and 0.12 ± 0.03 APE on day 8.

##### Muscle preparation to determine myofibrillar fractional synthesis rate

Frozen muscle samples (40 to 60 mg) were homogenized in 500 µl of ice‐cold Tris homogenization buffer (25 mm Tris‐HCl, TritonX‐100 (0.5% final volume), protease inhibitor (catalogue no. 4693116001; Roche, Basel Switzerland) and phosphatase inhibitor (catalogue no. 4906845001; Roche). Metal beads were added to all samples and run at 20 Hz for 40 s in a tissue homogenizer (Tissue Lyser II; Qiagen, Valencia, CA, USA). The muscle homogenate was then centrifuged at 4500 rpm for 10 min at 4°C. The supernatant was removed and the pellet was used to prepare the myofibrillar fraction as previously described (Burd *et al*. 2010). Cation exchange chromatography was then performed on the myofibrillar samples using columns containing Dowex resin (Dowex 50wx8‐200 ion exchange resin; Sigma‐Aldrich, St Louis, MO, USA) to extract free amino acids from the myofibrillar fractions (Burd *et al*. 2010). The amino acid samples were then derivatized as their *N*‐acetyl‐*n*‐propyl‐esters, in accordance with previously reported protocols (Hector *et al*. 2018; McGlory *et al*. 2018).

##### FSR

The ^2^H/^1^H ratio of the myofibrillar samples were determined using gas chromatography pyrolysis isotope ratio mass spectrometry (Metabolic Solutions, Nashua, NH, USA), to assess the incorporation of deuterium into protein‐bound alanine. This was used to assess the FSR of myofibrillar proteins with the use of the enrichment of body water, corrected for the mean number of deuterium moieties incorporated per alanine (i.e. 3.7), as described previously (Wilkinson *et al*. 2014), as the surrogate precursor labelling between subsequent biopsies. Saliva enrichments were assessed using cavity ring‐down spectroscopy using methods, as described previously (Hector *et al*. 2018; McGlory *et al*. 2018).

The standard equation (Wilkinson *et al*. 2014) used to determine FSR was:

$$\mathrm{FSR}\;\left(\%\;\mathrm{da}y^{-1}\right)=\left(\left(E_{t1}-E_{t0}\right)/\left(E_{p}\times time\right)\right)\times 100$$

where FSR is the fractional synthetic rate, *E*_t1_ is APE day 8, *E*_t0_ is APE day 3, *E*_p_ is average saliva APE, time is time between biopsies (days) and APE is the atomic percentage excess.

#### Preparation of whole‐muscle lysates for western blotting

Frozen muscle (10–20 mg) was homogenized as described previously (Granata *et al*. 2016) in an ice‐cold lysis buffer (dilution 1:20 w/v) containing 50 mm Tris, 150 mm NaCl, 1 mm EDTA, 1% IGEPAL, deionized water and a protease/phosphatase inhibitor cocktail (Cell Signaling Technology, Danvers, MA, USA), adjusted to pH 7.4. Protein concentration was determined in triplicate with a commercial colorimetric assay (Protein Assay kit‐II; Bio‐Rad, Gladesville, NSW, Australia), against bovine serum albumin standards (A9647; Sigma‐Aldrich).

#### Western blotting

Muscle homogenate was diluted in 4 × Laemmli buffer (0.25 m Tris, 4% SDS, 20% glycerol, 0.015% bromophenol blue and 10% 2‐mercaptoethanol) and equal amounts of total protein (15 or 20 µg) were loaded in different wells on Criterion™ 4–20% TGX Stain‐Free™ Precast Gels (Bio‐Rad). A stain‐free system (Bio‐Rad, Australia) was used as a loading control, with protein expression normalized to total protein loaded per lane. Each participant's samples were loaded into adjacent lanes on the same gel. Each gel also contained four to six internal standards of varying dilutions, made from a mixed homogenate of every sample in equal concentrations. These standards were used to form a calibration curve, with density plotted against protein content. Protein abundance was then calculated from the measured band intensity for each sample on the gel, using the linear regression equation from the calibration curve (Murphy & Lamb, 2013).

Gel electrophoresis was run for 20 min at 80 V and then for a further 60 to 90 min at 80 to 150 V. Transfer of proteins from the gel to 0.2 µm polyvinylidene fluoride membrane at 25 V for 10 min was performed via turbo transfer (Bio‐Rad). Membranes were then blocked in 5% non‐fat dry milk diluted in Tris‐buffered saline with 0.1% Tween‐20 (TBST) for 60 min. Membranes were washed in TBST and incubated overnight at 4°C with the appropriate primary antibody, prepared at a 1:1000 dilution in TBST with 5% bovine serum albumin and 0.02% sodium azide. The primary antibodies used were from Cell Signaling Technologies and include total AKT (CST9272), p‐AKT^ser473^ (CST9271), total mTOR (CST2983), p‐mTOR^ser2448^ (CST5586), caspase‐3 (CST9662), total p70S6K (CST9202), total TSC2 (CST4308), p‐TSC2^thr1462^ (CST3617), LC3 (CST3868), p‐4EBP1^thr37/46^ (CST2855) and NFκB (CST8242). The measurement of p‐p70S6K^thr389^ protein content could not be assessed because of issues with respect to obtaining reliable results with the available antibodies (i.e. CST9205 and CST9234). Following four TBST washes, the membranes were then incubated at room temperature with the appropriate host species‐specific secondary antibody for 60 min, before being exposed to a chemiluminescence solution (Clarity Western ECL; Bio‐Rad; or Supersignal West Femto Maximum Sensitivity Substrate; Thermo Fisher, Waltham, MA, USA). Images were taken with a ChemiDoc Imaging System fitted (Bio‐Rad). Densitometry was performed with Image Lab, version 5.0 (Bio‐Rad). Images are typically displayed with at least five bandwidths above and below the band of interest.

#### Quantitative RT‐PCR

##### RNA extraction

RNA was extracted from frozen muscle samples (10–20 mg) by adding 800 µL of TRIzol (15596‐026; Life Technologies, Grand Island, NY, USA) and a stainless steel metal bead to each sample and using a homogenizing instrument (Tissue Lyser II; Qiagen) (Kuang *et al*. 2018). Prior to storage, separate aliquots were taken for RNA quantification using a Nanodrop spectrophotometer (Thermo Fisher) and RNA integrity testing using an automated microcapillary electrophoresis system (Experion; Bio‐Rad Laboratories, Hercules, CA, USA). cDNA was synthesized from 1 µg of RNA using the iScript™ Reverse transcription supermix for RT‐PCR kit (Bio‐Rad Laboratories). DEPC water (180 µL) was added to all samples following cDNA synthesis and samples were stored at −20°C. As a result of small muscle sample collections for one of the participants, only samples from seven participants in the NS group were prepared for RT‐PCR. Relative mRNA expression was measured by qPCR (QuantStudio 7 Flex; Applied Biosystems, Foster City, CA, USA) using SsoAdvanced Universal SYBR Green Supermix (Bio‐Rad). Primers were designed using Primer‐BLAST (Ye *et al*. 2012) to include all splice variants, and were purchased from Sigma‐Aldrich. All reactions were performed in duplicate on 384‐well MicroAmp optical plates (4309849; Applied Biosystems) using an epMotion M5073 automated pipetting system (Eppendorf AG, Hamburg, Germany). The total reaction volume of 5 µL contained cDNA template, 2.5 µL of 2 × mastermix and 0.3 µm or 0.9 µm primers. All assays ran for 10 min at 95°C, followed by 40 cycles of 15 s at 95°C and 60 s at 60°C.

##### Primer design and data normalization

The expression of each target gene was normalized to the geometric mean of expression of the three most stably expressed reference genes, as described previously (Vandesompele *et al*. 2002; Kuang *et al*. 2018), and using the 2^−ΔΔCt^ method (where Ct is the quantification cycle) (Schmittgen & Livak, 2008). A list of housekeeping and target gene primer sequences is provided in Table 3.

**Table 3:** RT‐PCR primer sequences

Primer name	Primer sequence	Product Size (bp)	Efficiency (%)	Accession number
Target genes				
*Myostatin*	F – GGAGAAGATGGGCTGAATCCG R – GCATCGTGATTCTGTTGAGTGC	111	98.9	NM_005259
*Murf1*	F – CCGTCGAGTGACCAAGGAGA R – CCAGGATGGCATACAACGTG	80	98.6	NM_032588
*Mafbx*	F – GCAGCTGAACAACATTCAGATCAC R – CAGCCTCTGCATGATGTTCAGT	97	99.5	NM_058229
*Foxo1*	F – TGAGGGTTAGTGAGCAGGTTAC R – GGACTGCTTCTCTCAGTTCCT	73	108.1	NM_002015
*Foxo3*	F –TTGGTTTGAACGTGGGGAAC R –TGTGTCAGTTTGAGGGTCTGC	119	91.1	NM_001455.4
*Mighty*	F – CCAACTCCGGAGCAAATTTTTCA R – TCCGAAGCACAAGCTTCACT	106	94.7	NM_024595
*p62/SQSTM1*	F – AGAATCAGCTTCTGGTCCATCGG R – CTTTTCCCTCCGTGCTCCAC	129	103.2	http://NM_003900.5
Housekeeping genes				
*B2M*	F – TGCTGTCTCCATGTTTGATGTATCT R – TCTCTGCTCCCCACCTCTAAGT	86	98	NM_004048.2
*ACTB*	F – GAGCACAGAGCCTCGCCTTT R – TCATCATCCATGGTGAGCTGGC	70	107	NM_001101.3
*TBP*	F – CAGTGACCCAGCAGCATCACT R – AGGCCAAGCCCTGAGCGTAA	205	99	NM_003194.4

F, forward primer; R, reverse primer.

### Statistical analysis

Statistical analyses were conducted using Prism, version 7.03 (GraphPad Software Inc., San Diego, CA, USA). Pre‐ to post‐intervention changes in gene expression and protein content were assessed for each group using a mixed ANOVA with one between‐subjects measure (group) and a within‐subjects measure (time). Significant effects of interaction (group × time), time (pre *vs*. post) and group (NS *vs*. SR *vs*. SR+EX) are reported where effects are seen. Where significant effects occurred, Bonferonni *post hoc* testing was performed to locate the differences. All statistical analyses of gene expression and protein content data were conducted using raw values. Gene expression data in‐text are reported as percent fold‐changes from pre‐intervention values (%, mean ± SD) with 95% confidence interval (CI) from fold‐change data (as a percentage), with the *P* value from the raw data reported. Data in the figures represent fold‐changes from pre‐intervention values, with individual responses. A one‐way ANOVA was used to assess differences between groups for the mean actigraphy sleep data and MyoPS data. All data in the text, figures and tables are presented as the mean ± SD, and 95% CI with *P* < 0.05 indicate statistical significance.

## Results

### Sleep data

There were no between‐group differences for baseline TST [mean ± SD TST (min) of nights 1 and 2, *P* value, NS; 448 ± 25 min, SR; 452 ± 17 min, and SR+EX; 459 ± 9 min, *P *= 0.50]. The NS group showed no difference in TST between the baseline and intervention periods [mean ± SD difference (min), 95% CI min, *P* value, 1 ± 28 min, 95% CI = –20 to 17 min, *P *> 0.99]. Both sleep‐restricted groups had a reduction in TST during their respective interventions compared to the baseline period (SR; –224 ± 20 min, 95% CI = −241 to −203 min, *P *< 0.001; SR+EX −223 ± 9 min, 95% CI = −242 to −204 min, *P *< 0.001). Compared to the NS group, TST during the intervention period was significantly reduced in both the SR group (−219 ± 8 min, 95% CI = −236 to −202 min, *P *< 0.001) and SR+EX group (−214 ± 8 min, 95% CI = −231.5 to −197 min, *P *< 0.001). There were no significant differences in TST between the SR and SR+EX groups during the intervention period (−5 ± 3 min, 95% CI = −23 to 12 min, *P *= 0.72).

### MyoPS

**Figure 2:**
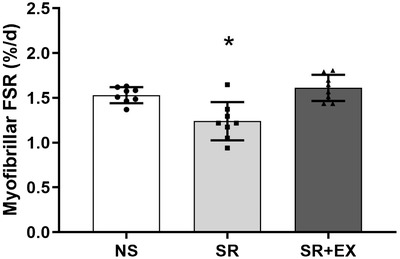
FSR of myofibrillar proteins throughout the sleep intervention NS, SR and SR+EX groups. Bars are the mean ± SD. *Significant difference from both NS and SR+EX conditions; one‐way ANOVA (*P* < 0.05).

There was a significant difference between groups for MyoPS throughout the intervention period (*P *< 0.001). Compared to the NS group, the myofibrillar protein FSR was significantly lower [mean ± SD between‐group difference FSR (% day^–1^), 95% CI, *P* value] in the SR group; –0.29 ± 0.08 FSR % day^–1^, 95% CI [−0.50, −0.09 FSR % day^–1^], *P *= 0.004) (Fig. 2). Furthermore, MyoPS was significantly lower in the SR group compared to the SR+EX group (−0.37 ± 0.09 FSR % day^–1^, 95% CI = −0.58 to −0.17 FSR % day^–1^, *P *< 0.001). There was no difference in MyoPS between the NS and SR+EX groups (0.08 ± 0.06 FSR % day^–1^, 95% CI = −0.29 to 0.12 FSR % day^–1^, *P *= 0.95).

### Protein synthesis and protein degradation related mRNA gene expression

**Figure 3:**
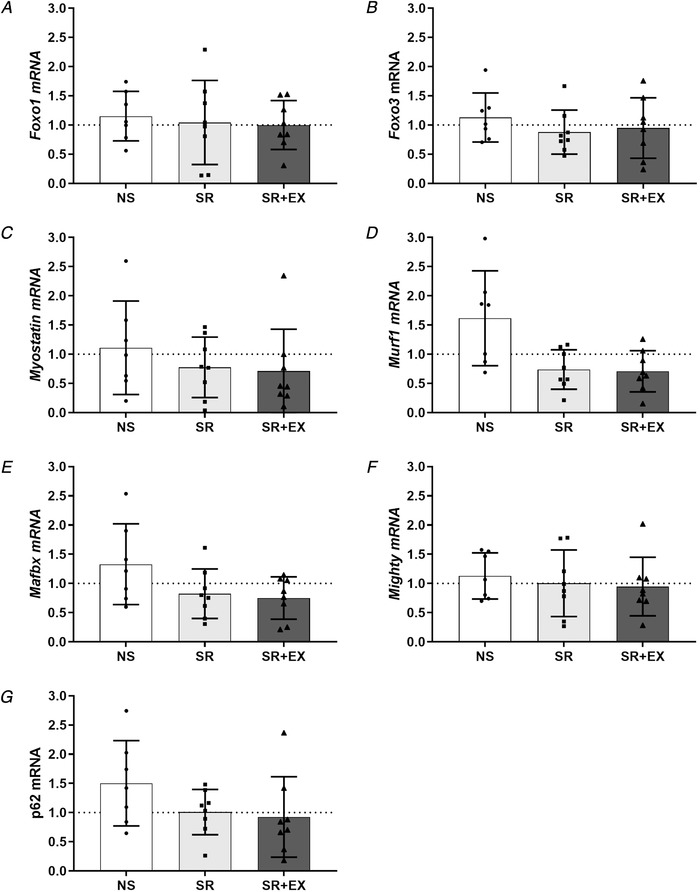
Skeletal muscle mRNA expression of genes related to protein synthesis, degradation and autophagy signalling pathways, normalized to pre‐intervention values (i.e. values shown are fold change from pre‐intervention, calculated via the 2^−ΔΔCt^ method). Foxo1 (A), Foxo3 (B), Myostatin (C), Murf1 (D), Mafbx (E), Mighty (F) NS, SR and SR+EX groups. Bars are the mean ± SD; *n *= 7 in NS, *n* = 8 in both SR and SR+EX. Individual participant responses are indicated by individual symbols.

There was no significant interaction effect for mRNA gene expression of *Foxo1* (*P *= 0.69), *Foxo3* (*P *= 0.74), *Myostatin* (*P *= 0.19), *Mafbx* (*P *= 0.24), *Mighty* (*P *= 0.81) or *p62/SQSTM1 (P *= 0.09) compared to pre‐intervention values. Despite there being a significant interaction effect for *Murf1* mRNA gene expression (*P *= 0.026), *post hoc* analyses revealed no difference between groups from pre to post‐intervention (NS: 61 ± 81%, 95% CI = −114 to −9%, *P *= 0.25), (SR: −26 ± 33%, 95% CI = −23 to 75%, *P *= 0.27), (SR+EX: −29 ± 35%, 95% CI = −20 to 28%, *P *= 0.20) (Fig. 3).

### Protein synthesis and protein degradation related protein content

**Figure 4:**
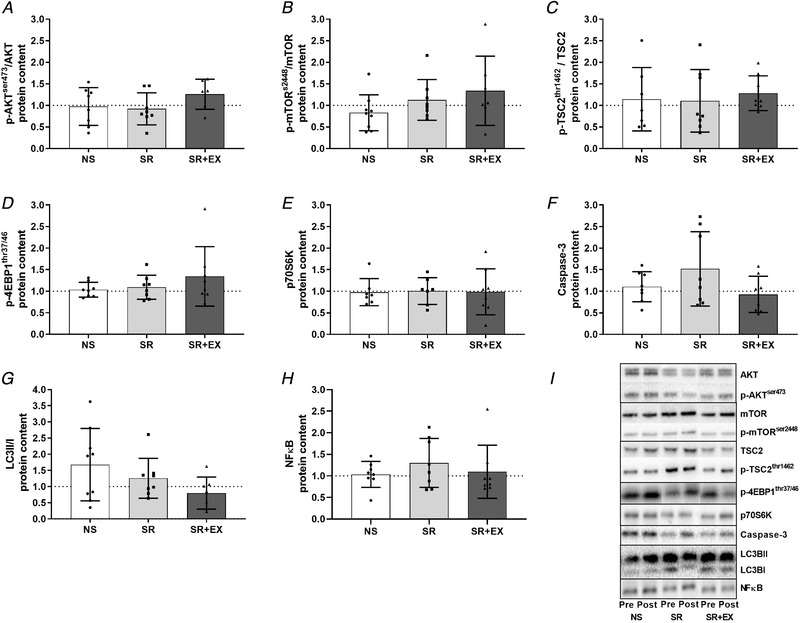
Skeletal muscle protein content of protein synthesis, autophagy and protein degradation pathways p‐AKT^ser473^/AKT (total) (*A*), p‐mTOR^ser2448^/mTOR (total) (*B*), p‐TSC2^thr462^/TSC2 (total) (*C*), p‐4EBP1^thr37/46^ (*D*), p70S6K (total) (*E*), Caspase‐3 (*F*), LC3BII/LC3BI ratio (*G*), total NFκB (*H*) and representative western blots images (*I*). Data are presented as the fold‐change from pre‐intervention. NS, SR and SR+EX groups. Bars are the mean ± SD; *n* = 8 in each group. Individual participant responses are indicated by individual symbols.

There were no changes in p‐AKT^ser473^/AKT (total) (*P *= 0.15), p‐mTOR^ser2448^/mTOR (Total) (*P* = 0.15), p70S6K (*P *= 0.28), Caspase 3 (*P *= 0.19), p‐4EBP1^thr37/46^ (*P *= 0.67), p‐TSC2^thr1462^/TSC2 (Total) (*P *= 0.29), LC3BII:I ratio (*P* = 0.88) or NFκB (*P *= 0.72) protein content from pre‐ to post‐intervention (Fig. 4).

## Discussion

We observed a significantly lower rate of MyoPS following 5 nights of SR, whereas there was no difference in rates of MyoPS between the SR+HIIE and NS group. There were, however, no changes in molecular markers that are commonly used to indicate activation of the protein synthesis and degradation pathways. These results provide novel insights into mechanisms underlying the previously described decreases in muscle mass that have been attributed to inadequate sleep, and suggest HIIE as a potential therapeutic intervention to mitigate these changes.

### Sleep restriction, exercise and MyoPS

Rates of protein synthesis are considered the primary driving mechanism for changes in muscle mass (atrophy and hypertrophy) (Rennie, 1985; Gibson *et al*. 1987; de Boer *et al*. 2007). Previous reports have shown reductions in muscle mass when obtaining insufficient sleep (Nedeltcheva *et al*. 2010; Dattilo *et al*. 2012; de Sa Souza *et al*. 2016); however, the present study is the first to demonstrate a lower rate of MyoPS in a SR group following 4 h TIB for five consecutive nights. This period of time was probably too short to detect any meaningful reductions in muscle mass or muscle fibre cross‐sectional area (CSA). However, persistent reductions in MyoPS are associated with the loss of muscle mass, as have been reported with a variety of muscle disuse conditions (i.e. step reduction, limb immobilization and bed rest) (de Boer *et al*. 2007; Breen *et al*. 2013). As such, the lower rate of MyoPS reported here aligns with observations of decreases in muscle fibre CSA, reported previously in rodents (following 96 h of sleep deprivation) (Dattilo *et al*. 2012; Monico‐Neto *et al*. 2015*a*; de Sa Souza *et al*. 2016) and increased loss of muscle mass with sleep restriction in humans (Nedeltcheva *et al*. 2010). Nonetheless, further research is needed to determine the long‐term effects of sleep restriction on both MyoPS and the maintenance of muscle mass as there may be implications for those populations at risk of regularly experiencing inadequate or reduced sleep (e.g. elderly populations, shift‐workers) or those interested in the maintenance of rates of MyoPS (e.g. athletes) (Akerstedt & Wright, 2009; Chien *et al*. 2015; Fullagar *et al*. 2016). Previous interventions where MyoPS is reduced in response to step reduction have suggested the development of an ‘anabolic resistance’, such that the amino acid‐induced stimulation of MyoPS is blunted (Breen *et al*. 2013). Because the SR group had rates of MyoPS that were ∼19% lower than that of the NS or SR+HIIE groups, it may be proposed that sleep restriction can inhibit MyoPS. As such, further research should investigate the potential effect sleep restriction may have on the anabolic stimuli provided by interventions such as feeding and exercise.

Resistance exercise is well known to increase rates of MyoPS in skeletal muscle with subsequent increases in muscle mass suggesting this exercise modality warrants further investigation in the context of sleep loss (Konopka & Harber, 2014; Brook *et al*. 2015; Brook *et al*. 2016; Robinson *et al*. 2017). However, increases in MyoPS (which may also be associated with skeletal muscle remodelling) have also been observed with endurance exercise (and in particular, HIIE), although the findings appear to be somewhat protocol‐dependent (Di Donato *et al*. 2014; Konopka & Harber, 2014). The results of the present study demonstrate that the rate of MyoPS was higher in the SR+EX group compared to the SR group. The higher MyoPS observed in the SR+EX group compared to the SR group is probably explained by aerobic exercise induced increases in MyoPS (Miller *et al*. 2005; Di Donato *et al*. 2014; Bell *et al*. 2015). Indeed, Miller *et al*. (2005) reported increases in MyoPS following 1 h of single‐leg kicking (67% *W*_max_), which remained elevated at 48 and 72 h post‐exercise. An additional group that combined normal sleep (8 h TIB) with exercise (i.e. NS+EX) would have allowed us to determine whether sleep restriction may have attenuated additional exercise‐induced‐increases in MyoPS similar to those reported previously with HIIE interventions (Bell *et al*. 2015). However, the comparable rates of MyoPS in the SR+EX and NS groups support previous findings that implicate HIIE as a potent stimulus for MyoPS.

### Molecular markers of protein turnover

In the present study, there were no detectable changes in mRNA expression or protein content of markers of either the protein synthesis or degradation signalling pathways (i.e. AKT‐mTOR‐p70S6K, UPS and autophagy pathways). Similar to the present study, no change in key regulators of the protein synthesis pathway (i.e. p‐mTOR^ser2448^ or p‐p70S6K^thr389^) were reported following 96 h of sleep deprivation in rats, despite reductions in muscle fibre CSA (Monico‐Neto *et al*. 2015*a*; de Sa Souza *et al*. 2016). By contrast to our findings, however, these same studies reported an increase in markers of the UPS and autophagy signalling pathways (i.e. p‐FOXO3, LC3 and p62/SQSTM protein content) (Monico‐Neto *et al*. 2015*a*; de Sa Souza *et al*. 2016) indicating that sleep deprivation increases MPB in rats. Although some evidence supports corresponding changes in MyoPS and its associated signalling responses (Brook *et al*. 2015), dissociations similar to those reported in the present study are not uncommon (de Boer *et al*. 2007; Greenhaff *et al*. 2008; Atherton *et al*. 2010). Furthermore, the absence of changes to the MPS and MPB signalling pathways may be explained by the transient nature of their regulation and in relation to biopsy timing, which has previously been demonstrated in different contexts (de Boer *et al*. 2007; Atherton *et al*. 2010). Despite not detecting changes to markers of the MPS and MPB signalling pathways in the present study, the D_2_O method for assessing MyoPS allows for an integrated measure of MyoPS (i.e. it represents MyoPS in the fed, fasted, exercised, sleep and awaken states) throughout the intervention, and also encapsulates the influence of sleep restriction and exercise in a realistic, free‐living setting, over an extended duration.

In the context of exercise, several studies have reported increases in phosphorylation of AKT^ser473^, mTOR^ser2448^ and decreases in eEF2^thr56^ protein, immediately following an endurance exercise session, with levels returning to baseline within 3–4.5 h (Mascher *et al*. 2007; Di Donato *et al*. 2014). Furthermore, although MPB increases following resistance exercise, it returns to resting values within 48 h (Phillips *et al*. 1997; Kumar *et al*. 2009). Given our post‐intervention biopsy occurred 48 h following the final exercise session, any changes in signalling markers may no longer have been detectable. Indeed, the present study showed that, even in the exercise group, there were no changes to MPS and MPB signalling. When taken together with previous literature, this probably means that changes in the molecular signalling pathways for MPS and MPB occur during the earlier stages of the sleep intervention or accumulated throughout the day as the homeostatic drive for sleep increased (rather than the hours immediately following sleep) and/or in the hours immediately following HIIE (de Boer *et al*. 2007; Greenhaff *et al*. 2008; Atherton *et al*. 2010). To fully determine the mechanisms that may underpin the lower rates of MyoPS observed in the SR group, and the comparable rates of MyoPS in the SR+EX and NS groups, further research is required with additional muscle sampling throughout the intervention to capture early signalling responses to exercise, feeding and changes as the duration of wakefulness is extended.

Given the capacity for HIIE to promote MyoPS (Di Donato *et al*. 2014; Bell *et al*. 2015) and the findings of the present study suggesting that HIIE may be capable of maintaining regular rates of MyoPS during periods of sleep restriction, collectively, these data may be of interest to populations for whom inadequate sleep is common, and the maintenance of muscle mass is of a particular concern (e.g. shift‐workers, athletic populations, military personnel) (Akerstedt & Wright, 2009; Fullagar *et al*. 2016; Mysliwiec *et al*. 2016). Furthermore, the prevalence of sarcopenia in older adults (>65 years of age) is increased in those who report sleeping <6 h per night (Chien *et al*. 2015; Buchmann *et al*. 2016; Hu *et al*. 2017). Although still speculative from the present acute data, the results of the present study suggest that regular HIIE may be used to preserve muscle mass and function in these populations and provides the foundation for future research in the area.

### Limitations

Although previous research suggests that alterations to MyoPS underlie changes in muscle mass (Rennie, 1985; Gibson *et al*. 1987; de Boer *et al*. 2007), the present study did not employ direct measurements of changes in muscle mass. Previous research has observed dual‐energy X‐ray absorptiometry‐derived losses in fat‐free soft tissue mass across a 14‐day sleep restriction period, although the magnitude of this effect was probably enhanced by the calorie‐restricted diet employed (Nedeltcheva *et al*. 2010). Nevertheless, the current intervention duration was probably too short to detect changes in lean mass with dual‐energy X‐ray absorptiometry (given the variability of the measure) (Bone *et al*., 2017) and future investigations of similar durations should consider incorporating direct measures of muscle mass (i.e. with magnetic resonance imaging or CSA). Although our data report a significantly lower rate of MyoPS (∼19%) with sleep restriction, with the present study design, it is unclear how this compares to baseline rates of MyoPS. As such, future studies may incorporate a baseline measurement into the study as a comparison measurement. Furthermore, as eluded to previously, the addition of a normal sleep and exercise group may have provided further mechanistic insight into the effect of sleep restriction and HIIE on MyoPS. Finally, without further research that includes female and older participants, the generalizability of these findings for the wider population remains to be elucidated.

## Conclusions

We discovered that, following five nights of sleep restriction (4 h TIB, each night), the rate of MyoPS was significantly lower (by 19%) compared to the NS and SR+EX groups. To our knowledge, this is the first observation of a sleep‐restriction‐induced change in MyoPS in humans. Importantly, we showed that participants in the SR+EX group had higher rates of MyoPS than the SR group and were similar to those in the NS group. However, there was a dissociation between the changes in MyoPS and the molecular signalling pathways that regulate protein synthesis and degradation. Although further investigations are needed, these findings suggest that sleep restriction can have detrimental effects on the processes that maintain muscle mass, via the potential suppression of MyoPS. Our findings may partly help to explain previous reports of reduced muscle mass in those experiencing insufficient sleep (Nedeltcheva *et al*. 2010). Our observation that those participants performing HIIE during the SR intervention were able to maintain their rates of MyoPS at levels comparable to the NS group could have important implications for a range of populations experiencing inadequate sleep and lends support to the idea that HIIE could be a viable intervention to mitigate certain detrimental effects associated with sleep loss.

## Additional information

### Competing interests

The authors declare that they have no competing interests.

### Author contributions

NS, GR, SP, DB and JB were involved in the conception and design of the present study. NS, ML, NP, AG, TS, JK, SP, DB and JB were involved in the acquisition, analysis or interpretation of the data. NS, ML, NP, AG, GR, TS, JK, SP, DB and JB were involved in drafting the work and revising it critically for important intellectual content. All authors approved the final version of the manuscript submitted for publication. All authors agree to be accountable for all aspects of the work in ensuring that questions related to the accuracy or integrity of any part of the work are appropriately investigated and resolved. All persons designated as authors qualify for authorship, and all those who qualify for authorship are listed.

### Funding

This publication was supported in part by a Sports Medicine Australia (SMA) Research Foundation Grant and Australian Postgraduate Award PhD Scholarship to NS. SMP gratefully acknowledges support from the Canada Research Chairs program and the funding for this work provided by the National Science and Engineering Research Council (NSERC) of Canada. TS was supported by an NSERC of Canada graduate scholarship.

## Supporting information


**Statistical Summary Document**
