# Supplementary material for: The effect of sleep restriction, with or without high‐intensity interval exercise, on myofibrillar protein synthesis in healthy young men
Source: J Physiol. 2020 Mar 11;598(8):1523–36. doi: 10.1113/JP278828 (PMC7217042; doi:10.1113/JP278828)
Supplement: Supplementary file 1 — Statistical Summary Document [file TJP-598-1523-s001.docx]

# Manuscript Title: The Effect of Sleep Restriction, With or Without Exercise, on Myofibrillar Protein Synthesis in Healthy Young Men

**Authors:** Nicholas J Saner, Matthew J-C Lee, Nathan W Pitchford, Jujiao Kuang, Gregory D Roach, Andrew Garnham, Tanner Stokes, Stuart M Phillips, David J Bishop, Jonathan D Bartlett.

**Animal model used, if applicable:**

**Underlying hypothesis:**

We hypothesised that (1) the rate of MyoPS would be lower in the sleep restriction group and this would be reflected by changes in the molecular signalling pathways associated with (2) MPS and (3) MPB, but performing three sessions of HIIE would attenuate this effect

**Definitions of ‘n’:**

Twenty-four healthy, recreationally-active men, aged between 18 and 40 years of age, volunteered to participate, with Normal Sleep group (NS, *n*=8), Sleep Restriction group (SR, *n*=8) and Sleep Restriction and Exercise group (SR+EX, *n*=8).

**Statistical summary table:**

| Experimental question number* | Finding/ conclusion | Experimental location/ variable  e.g. cortex vs cerebellum or genotype | Mean value  (or other summary statistic) | SD | n (value) | P** | Units | Data comparisons  e.g. WT vs KO | Statistical test | Any other variable  e.g. subjects’ age or sex | Figure/table in which data are presented | Comments  e.g. observation |
| --- | --- | --- | --- | --- | --- | --- | --- | --- | --- | --- | --- | --- |
| 1 MyoPS | There was a significant difference between groups for MyoPS throughout the intervention period | Myofibrillar protein synthesis (skeletal muscle) | SR = 1.24  NS = 1.53  SR+EX = 1.61 | 0.21  0.09  0.14 | 24 (8 in each group | **<0.001** | FSR % | NS vs SR vs SR+EX | One-way ANOVA |  | Figure 2 | Interaction effect |
| 1 MyoPS | Myofibrillar protein fractional synthetic rate (FSR) was significantly lower in SR compared to NS | Myofibrillar protein synthesis (skeletal muscle) | -0.29  Mean difference | 0.08 | 16 | **0.004** | FSR% | NS vs SR | One-way ANOVA |  | Figure 2 | Bonferroni post hoc  CI [-0.50, -0.09 FSR %/day] |
| 1 MyoPS | Myofibrillar protein fractional synthetic rate (FSR) was significantly lower in SR compared to SR+EX | Myofibrillar protein synthesis (skeletal muscle) | -0.37  Mean difference | 0.09 | 16 | **<0.001** | FSR% | SR vs SR+EX | One-way ANOVA |  | Figure 2 | Bonferroni post hoc  CI [-0.58, -0.17 FSR %/day] |
| 1 MyoPS | Myofibrillar protein fractional synthetic rate (FSR) was similar between NS and SR+EX | Myofibrillar protein synthesis (skeletal muscle) | 0.08  Mean difference | 0.06 | 16 | 0.95 | FSR% | NS vs SR+EX | One-way ANOVA |  | Figure 2 | Bonferroni post hoc  CI [-0.29, 0.12 FSR%/day] |
| 2 MPS molecular markers | There was no significant effect between groups for p-AKT between pre- and post-intervention | AKT phosphorylation (skeletal muscle) | SR = 0.92  NS = 0.98  SR+EX = 1.26 | 0.37  0.44  0.35 | 8  8  6 | 0.15 | AU | NS vs SR vs SR+EX | ANOVA |  | Figure 4 | Interaction effect |
| 2 MPS molecular markers | There was no significant effect between groups for p-mTOR between pre- and post-intervention | mTOR phosphorylation (skeletal muscle) | SR = 1.12  NS = 0.88  SR+EX = 1.34 | 0.47  0.41  0.80 | 8  8  7 | 0.15 | AU | NS vs SR vs SR+EX | ANOVA |  | Figure 4 | Interaction effect |
| 2 MPS molecular markers | There was no significant effect between groups for p-TSC2 between pre- and post-intervention | TSC2 phosphorylation (skeletal muscle) | SR = 1.11  NS = 1.14  SR+EX = 1.28 | 0.72  0.74  0.40 | 8  7  8 | 0.29 | AU | NS vs SR vs SR+EX | ANOVA |  | Figure 4 | Interaction effect |
| 2 MPS molecular markers | There was no significant effect between groups for p4EBP1 between pre- and post-intervention | 4EBP1 phosphorylation (skeletal muscle) | SR = 1.10  NS = 1.06  SR+EX = 1.34 | 0.28  0.16  0.69 | 8  7  7 | 0.67 | AU | NS vs SR vs SR+EX | ANOVA |  | Figure 4 | Interaction effect |
| 2 MPS molecular markers | There was no significant effect between groups for P70S6K protein between pre- and post-intervention | P70S6K protein (skeletal muscle) | SR = 1.00  NS = 0.98  SR+EX = 0.99 | 0.31  0.31  0.53 | 7  7  7 | 0.28 | AU | NS vs SR vs SR+EX | ANOVA |  | Figure 4 | Interaction effect |
| 2 MPS molecular markers | There was no significant effect between groups for caspase-3 protein between pre- and post-intervention | Caspase-3 protein (skeletal muscle) | SR = 1.52  NS = 1.11  SR+EX = 0.93 | 0.86  0.35  0.43 | 8  8  8 | 0.19 | AU | NS vs SR vs SR+EX | ANOVA |  | Figure 4 | Interaction effect |
| 2 MPS molecular markers | There was no significant effect between groups for LC3II/I protein between pre- and post-intervention | LC3II/I protein (skeletal muscle) | SR = 1.26  NS = 1.64  SR+EX = 0.80 | 0.62  1.19  0.49 | 8  8  7 | 0.19 | AU | NS vs SR vs SR+EX | ANOVA |  | Figure 4 | Interaction effect |
| 3 MPB molecular markers | There was no significant effect between groups for FOXO1 mRNA between pre- and post-intervention | FOXO1 gene (skeletal muscle) | SR = 1.04  NS = 1.15  SR+EX = 1.00 | 0.72  0.42  0.42 | 8  7  8 | 0.69 | AU | NS vs SR vs SR+EX | ANOVA |  | Figure 4 | Interaction effect |
| 3 MPB molecular markers | There was no significant effect between groups for FOXO3 mRNA between pre- and post-intervention | FOXO3 gene (skeletal muscle) | SR = 0.88  NS = 1.13  SR+EX = 0.95 | 0.38  0.42  0.52 | 8  7  8 | 0.74 | AU | NS vs SR vs SR+EX | ANOVA |  | Figure 4 | Interaction effect |
| 3 MPB molecular markers | There was no significant effect between groups for myostatin mRNA between pre- and post-intervention | Myostatin gene (skeletal muscle) | SR = 0.78  NS = 1.11  SR+EX = 0.71 | 0.51  0.80  0.72 | 8  7  8 | 0.19 | AU | NS vs SR vs SR+EX | ANOVA |  | Figure 4 | Interaction effect |
| 3 MPB molecular markers | There was no significant effect between groups for murf1 mRNA between pre- and post-intervention | Murf1 gene (skeletal muscle) | SR = 0.88  NS = 1.13  SR+EX = 0.95 | 0.38  0.42  0.52 | 8  7  8 | **0.026** | AU | NS vs SR vs SR+EX | ANOVA |  | Figure 4 | Interaction effect |
| 3 MPB molecular markers | There was no significant effect between groups for MafBx mRNA between pre- and post-intervention | Mafbx gene (skeletal muscle) | SR = 0.82  NS = 1.33  SR+EX = 0.75 | 0.42  0.69  0.36 | 8  7  8 | 0.24 | AU | NS vs SR vs SR+EX | ANOVA |  | Figure 4 | Interaction effect |
| 3 MPB molecular markers | There was no significant effect between groups for mighty mRNA between pre- and post-intervention | Mighty gene (skeletal muscle) | SR = 1.00  NS = 1.13  SR+EX = 0.95 | 0.57  0.40  0.50 | 8  7  8 | 0.81 | AU | NS vs SR vs SR+EX | ANOVA |  | Figure 4 | Interaction effect |
| 3 MPB molecular markers | There was no significant effect between groups for p62 mRNA between pre- and post-intervention | p62 gene (skeletal muscle) | SR = 1.00  NS = 1.50  SR+EX = 0.93 | 0.39  0.73  0.69 | 8  7  8 | 0.09 | AU | NS vs SR vs SR+EX | ANOVA |  | Figure 4 | Interaction effect |

*You may use multiple lines for the same question to indicate multiple comparisons

** Authors may wish to make the text bold where p is considered significant against a stated confidence limit
